# Assessing treatment adherence is crucial to determine adequacy of mineralocorticoid therapy

**DOI:** 10.1530/EC-23-0059

**Published:** 2023-08-02

**Authors:** Riccardo Pofi, Ilaria Bonaventura, Joanne Duffy, Zoe Maunsell, Brian Shine, Andrea M Isidori, Jeremy W Tomlinson

**Affiliations:** 1 Department of Endocrinology, Oxford Centre for Diabetes, Endocrinology and Metabolism and NIHR Oxford Biomedical Research Centre, Churchill Hospital, University of Oxford, Oxford, UK; 2 Department of Experimental Medicine, Sapienza University of Rome, Viale Regina Elena, Rome, Italy; 3 Department of Clinical Chemistry and Immunology, Heartlands Hospital, Birmingham, UK; 4 Department of Clinical Biochemistry, Oxford University Hospitals NHS Foundation Trust, Oxford, UK

**Keywords:** mineralocorticoids, glucocorticoids, congenital adrenal hyperplasia, Addison’s disease, renin

## Abstract

**Background:**

There is no consensus strategy for mineralocorticoid (MC) therapy titration in patients with primary adrenal insufficiency (PAI). We aim to measure serum fludrocortisone (sFC) and urine fludrocortisone (uFC) levels and to determine their utility, alongside clinical/biochemical variables and treatment adherence to guide MC replacement dose titration.

**Methods:**

Multi-centre, observational, cross-sectional study on 41 patients with PAI on MC replacement therapy. sFC and uFC levels (measured by liquid chromatography-tandem mass spectrometry), plasma renin concentration (PRC), electrolytes (Na^+^, K^+^), mean arterial blood pressure (MAP), total daily glucocorticoid (dGC) and MC (dMC) dose, and assessment of treatment adherence were incorporated into statistical models.

**Results:**

We observed a close relationship between sFC and uFC (*r* = 0.434, *P* = 0.005) and between sFC and the time from the last fludrocortisone dose (*r* = −0.355, *P* = 0.023). Total dMC dose was related to dGC dose (*r* = 0.556, *P* < 0.001), K^+^ (*r* = −0.388, *P* = 0.013) as well as sFC (*r* = 0.356, *P* = 0.022) and uFC (*r* = 0.531, *P* < 0.001). PRC was related to Na^+^ (*r* = 0.517, *P* < 0.001) and MAP (*r* = −0.427, *P* = 0.006), but not to MC dose, sFC or uFC. Regression analyses did not support a role for sFC, uFC or PRC measurements and confirmed K^+^ (*B = *−44.593, *P* = 0.005) as the most important variable to guide dMC titration. Of the patients, 32% were non-adherent with replacement therapy. When adherence was inserted into the regression model, it was the only factor affecting dMC.

**Conclusions:**

sFC and uFC levels are not helpful in guiding dMC titration. Treatment adherence impacts on clinical variables used to assess MC replacement and should be included as part of routine care in patients with PAI.

## Introduction

Primary adrenal insufficiency (PAI) is a rare disease consisting of the inability of the adrenal cortex to produce adequate levels of glucocorticoids (GCs), with or without concomitant inadequate production of mineralocorticoids (MCs) and adrenal androgens (1). The most common causes of PAI are autoimmune adrenalitis (Addison's disease, AD) (2) and congenital adrenal hyperplasia (CAH), a group of rare genetic disorders characterized by impaired or deficient activity in steroidogenic enzymes (3, 4). PAI is a potentially life-threatening condition with a significant risk of adrenal crisis and requires lifelong GC often with additional MC replacement therapy (5, 6, 7).

Fludrocortisone (FC) has been the most commonly prescribed MC since its discovery in 1954 (8). The pharmacokinetic characteristics of FC (9), alongside the ambition to mimic the physiological circadian secretion of aldosterone which is similar to that of cortisol (10), mean that it is usually taken once a day in the morning (11). Commonly prescribed daily doses range from 0.05 to 0.2 mg and are believed to provide adequate MC replacement in adults and adolescents with PAI (11). FC is rapidly absorbed and subsequently converted to 9a-fluorocortisol, the active metabolite of FC. The plasma peak of FC appears within 1–2 h of oral absorption and is eliminated principally through urinary excretion (12). It is the fluorine atom on carbon 9 of the FC molecule that provides protection itself through rapid conversion and inactivation by 11β-hydroxysteroid dehydrogenase type 2 in the kidney and thus ensures its continued ability to bind and activate the MC receptor, leading to sodium retention, potassium elimination and water reabsorption (8).

Much attention has focused on the optimization of GC replacement in patients with PAI, driven, at least in part, by the increasing awareness of the detrimental adverse effects of circulating GC excess alongside the development of novel GC preparations (13, 14, 15, 16, 17, 18, 19, 20, 21). In contrast, there has been comparatively little attention on MC replacement (12, 22, 23). Currently, the Endocrine Society’s guidelines recommend monitoring MC replacement through clinical assessment (salt craving, blood pressure or oedema) and measuring blood electrolytes and renin. General well-being, maintaining electrolytes in the normal range alongside a normal blood pressure without evidence of postural hypotension and achieving renin levels in the upper part of the reference range have been suggested as indicators of the adequacy of MC replacement (11). However, our previous analysis has suggested that routine measurements of plasma renin do not enhance the ability to titrate MC replacement dose in a precise manner, but that assessments of electrolytes, blood pressure and clinical symptoms remain important (24). There are relatively few published studies that have tried to develop strategies to optimize MC replacement, but these are relatively small, mostly retrospective and have shown conflicting results (22, 24, 25, 26, 27). Potential explanations underpinning these discrepancies include their retrospective study design and as a consequence, the inability to make an accurate assessment of treatment adherence, an important factor that is also frequently neglected in randomized controlled trials (28, 29). As a result, there is still a significant unmet clinical need to develop strategies that are able to facilitate the accurate titration of MC replacement.

Previous studies to examine the potential utility of measuring FC levels in serum and urine (sFC, uFC) alongside accurate assessments of treatment adherence to guide MC preplacement have not been performed. We performed an observational clinical study to determine if combining sFC and uFC measurements, treatment adherence assessment with other routine clinical parameters (including electrolytes, blood pressure and plasma renin) could help guide MC replacement dose titration.

## Methods

### Patient selection

We performed a multi-centre, observational, cross-sectional study on patients with PAI treated with GC and MC replacement therapy. Consecutive patients were enrolled from a dedicated endocrine outpatient clinic in two different referral centres (Oxford Centre for Diabetes Endocrinology and Metabolism, Churchill Hospital, Oxford, UK; V clinica medica, Policlinico Umberto I di Roma, Rome, Italy) from June 2019 until March 2020.

All the adult patients attending the dedicated outpatient endocrine clinics for patients with AD or CAH were asked to participate in the study. Data and samples were collected as part of ‘real-life’ clinic consultations and included: anthropometric parameters (age, sex, weight, height, body mass index (BMI), systolic and diastolic blood pressure); details on MC and GC replacement therapy (preparation, dose frequency and total daily dose) and other concomitant medications (including drugs interfering with RAA systems such as antihypertensives or diuretics); standard laboratory biochemical analyses including sodium, potassium and plasma renin concentration (PRC); blood and urine (spot) samples were collected for serum and urinary FC assays. Mean arterial blood pressure (MAP) was calculated as diastolic blood pressure + 1/3 of differential blood pressure (systolic blood pressure minus diastolic blood pressure). To ensure uniformity and comparability of data the collected data, all GC doses were expressed as hydrocortisone equivalent (HCeq) using standardised conversions (30).

Patient adherence with both GC and MC replacement was evaluated through specific questions asked to the patients (see supplementary data); patients were asked for the number of GC and MC treatment doses missed in the last week as well as the time since their last replacement dose administration. An indirect assessment of adherence was made through a comparison of measured FC levels relative to known pharmacokinetic parameters of FC and the recorded time and dose of administration for individual participants. Patients were defined as ‘non-adherent’ if they missed one or more doses of GC or MC replacement in the preceding 7 days.

### Plasma renin concentration assay

Two different assays were used for PRC measurement. In Rome, PRC was measured by immunoradiometric assay, using Beckman Coulter reagents (ref. DSL25100). Mouse monoclonal antibodies directed against two different epitopes of renin are used (sandwich-type assay). Samples or calibrators are incubated in tubes pre-coated with one monoclonal antibody, and with a second liquid ^125^I-labelled monoclonal antibody. After incubation, unbound reagents are removed by washing the tubes. The amount of ^125^I-labelled anti-renin bound to the tube is directly proportional to the concentration of renin present in the sample. A standard curve is constructed, and PRC is obtained from the curve by interpolation. EDTA plasma samples were collected and processed at room temperature, to avoid cryoactivation of prorenin, giving falsely high active renin levels. The measurement range (from analytical sensitivity to the highest calibrator) is 0.81–500 pg/mL. The high specificity of the assay was confirmed by extremely low or undetectable cross-reactivity with several molecules (plasmin, trypsin, cathepsin B, cathepsin D, ACE and albumin).

In Oxford, PRC was measured by automated immunoassay, using the IDS-iSYS Direct Renin assay (Immunodiagnostic Systems Limited, Boldon, Tyne and Wear, UK). Two monoclonal antibodies directed against two different epitopes of renin are used in the assay; one labelled with acridinium and the other labelled with biotin.

Samples, calibrators and controls are incubated with both antibodies in specific system buffers. Streptavidin-coated magnetic particles are added, and following further incubation, the particles (bound to the renin-antibody complexes) are ‘captured’ using a magnet. The particles then undergo a wash step and a ‘trigger’ reagent is added. The amount of light emitted by the acridinium label is directly proportional to the concentration of renin present in the original sample.

The IDS-iSYS assay utilises a two-point calibration, measuring three replicates of each level. A four-parameter logistic curve fit is used to calculate the renin concentrations. The measurement range of the assay is 1.8–550 mIU/L. Typical assay performance characteristics in terms of coefficient of variation are as follows: 6.0% at 15.5 mIU/L, 1.8% at 101.8 mIU/L and 1.9% at 327.5 mIU/L.

To ensure comparability across assays, all the PRC levels were expressed as mIU/mL, using 1.67 as conversion factors as previously described (31).

### Serum and urine fludrocortisone levels assay

sFC and uFC were measured following liquid–liquid extraction by isotope dilution liquid chromatography-tandem mass spectrometry. Deuterated internal standard (Fludrocortisone D5, Sigma) was added to each sample, QC and calibrator followed by diethylether. Samples were mixed and then placed into an ethanol cold bath and the sample was frozen. The top solvent layer was poured off into clean glass tubes which were then evaporated to dryness in a vacuum drier (Genevac, Warminster, PA, USA). Samples were reconstituted in 50:50 methanol:water and injected into a Shimadzu HPLC system coupled with an AB Sciex 5000 tandem mass spectrometer. A Hypersil GOLD 1.9 µm, 100 × 2.1 mm (Thermo Fisher) chromatography column and samples eluted using mobile phases ammonium acetate in water (A) and 0.2% formic acid in methanol (B). APCI ionisation and positive mode ionisation were used. FC was monitored using transition 381.2>343.2 and internal standard using 386.2>348.4.

### Statistical analysis

The distribution of continuous variables was tested using the Shapiro–Wilk’s test and is reported as mean and 95% confidence interval (95% CI) or median and interquartile range (IQR, 25th–75th percentile), as appropriate. Categorical variables are expressed as percentage and frequency. Linearity was established by visual inspection of a scatterplot.

As per the distribution of data, a Pearson’s product–moment or Spearman’s rank-order correlation was run to assess the baseline relationship between different variables; an independent sample t-test or a Mann–Whitney *U* test was run to determine possible differences in the outcome variables between groups. A multiple regression analysis was run to test the effects of selected variables on the likelihood of predicting the total daily MC dose. The level of statistical significance was set at a *P* value < 0.05. All statistical computations were conducted with the IBM SPSS Statistics (version 27, IBM) and Prism 7.0 software package (GraphPad Software, Inc.).

This study was conducted under the Declaration of Helsinki and good clinical practice. Informed consent has been obtained from each patient after a full explanation of the purpose and nature of all procedures used. The study was approved by the Trust audit team and conducted and registered as a local quality improvement (QI-8109).

## Results

Forty-one patients (61% male, median age 39 years, IQR 27–56) with PAI (58% with AD, 42% with SW-CAH) receiving stable daily GC (median 23 mg HCeq, IQR 20–32) and FC (median 100 μg, IQR 50–200) replacement therapy were consecutively recruited into the study (Table 1). Of the patients, 66% (*n* = 27) were treated with hydrocortisone (HC) as GC replacement (16 of whom received modified-release HC, MRHC), whereas 34% (*n* = 14) were treated with prednisolone. GC treatment regimens were as follows: 37% (*n* = 15) received GC as a single dose and 44% (*n* = 18) and 19% (*n* = 8) were prescribed with two (BID) or three (TID) doses, respectively. All patients were treated with FC: 88% (*n* = 36) received FC as a single dose and 10% (*n* = 4) and 2% (*n* = 1) were prescribed with TID and BID regimens, respectively. Patient characteristics are presented in Table 1.

**Table 1:** Characteristics of 41 patients with PAI enrolled into the study.

	Whole cohort (*n* = 41)	AD (*n* = 24)	SW-CAH (*n* = 17)
Age (years)	39 (27–56)	50 (33–71)	31 (26–37)
Male % (*n*)	61 (25)	62 (15)	59 (10)
Female % (*n*)	39 (16)	38 (9)	41 (7)
Total daily MC dose (μg/day)	100 (50–200)	50 (50–150)	200 (125–200)
Non-adherent to MC % (*n*)	12 (5)	8 (2)	18 (3)
Total daily GC dose (mg/day, HCeq)	23 (20–32)	22.5 (18–28)	28 (24–38)
Non-adherent to GC % (*n*)	29 (12)	8 (2)	59 (10)
Hydrocortisone % (*n*)	66 (27)	100 (24)	18 (3)
Prednisolone % (*n*)	34 (14)	/	82 (14)
BMI (kg/m²)	26.2 (21.2–30.3)	25.2 (21.2–29.4)	29.5 (20.9–33.1)
SBP (mmHg)	120 ± 16	116 ± 14	126 ± 17
DBP (mmHg)	72 ± 9	72 ± 8	72 ± 12
MAP (mmHg)	88 ± 10	86 ± 8	90 ± 12
Na⁺ (mmol/L)	140 (138–142)	140 (138–143)	140 (139–141)
K⁺ (mmol/L)	4.1 (3.6–4.6)	4.5 (3.9–5)	3.6 (3.4–4)
PRC (μIU/mL)	136.2 (42.8–310.8)	107.3 (39.8–251.7)	164.6 (64.2–406.8)
17-OH-progesterone (nmol/L)	/	/	35.1 (4.0–102.2)
Androstenedione (nmol/L)	/	/	9.6 (1.7–17.5)
sFC (pg/mL)	29.5 (19.5–47.6)	25.6 (14.5–62.2)	29.9 (23.1–40.6)
uFC (pg/mL)	127 (49.9–249.5)	190.5 (50.2–273.7)	95.6 (48.9–165.5)

Data are shown as mean ± s.d. or median (IQR) as appropriate for the distribution of data.

AD, Addison’s disease; BMI, body mass index; DBP, diastolic blood pressure; GC, glucocorticoid; HCeq, equivalent dose of hydrocortisone; K^+^, potassium levels; MAP, mean arterial pressure; MC, mineralocorticoid; Na^+^, sodium levels; PRC, plasma renin concentration; SBP, systolic blood pressure; sFC, serum fludrocortisone levels; SW-CAH, salt-wasting congenital adrenal hyperplasia; uFC, urinary fludrocortisone levels.

### Relationships between sFC, uFC and clinical parameters

**Figure 1:**
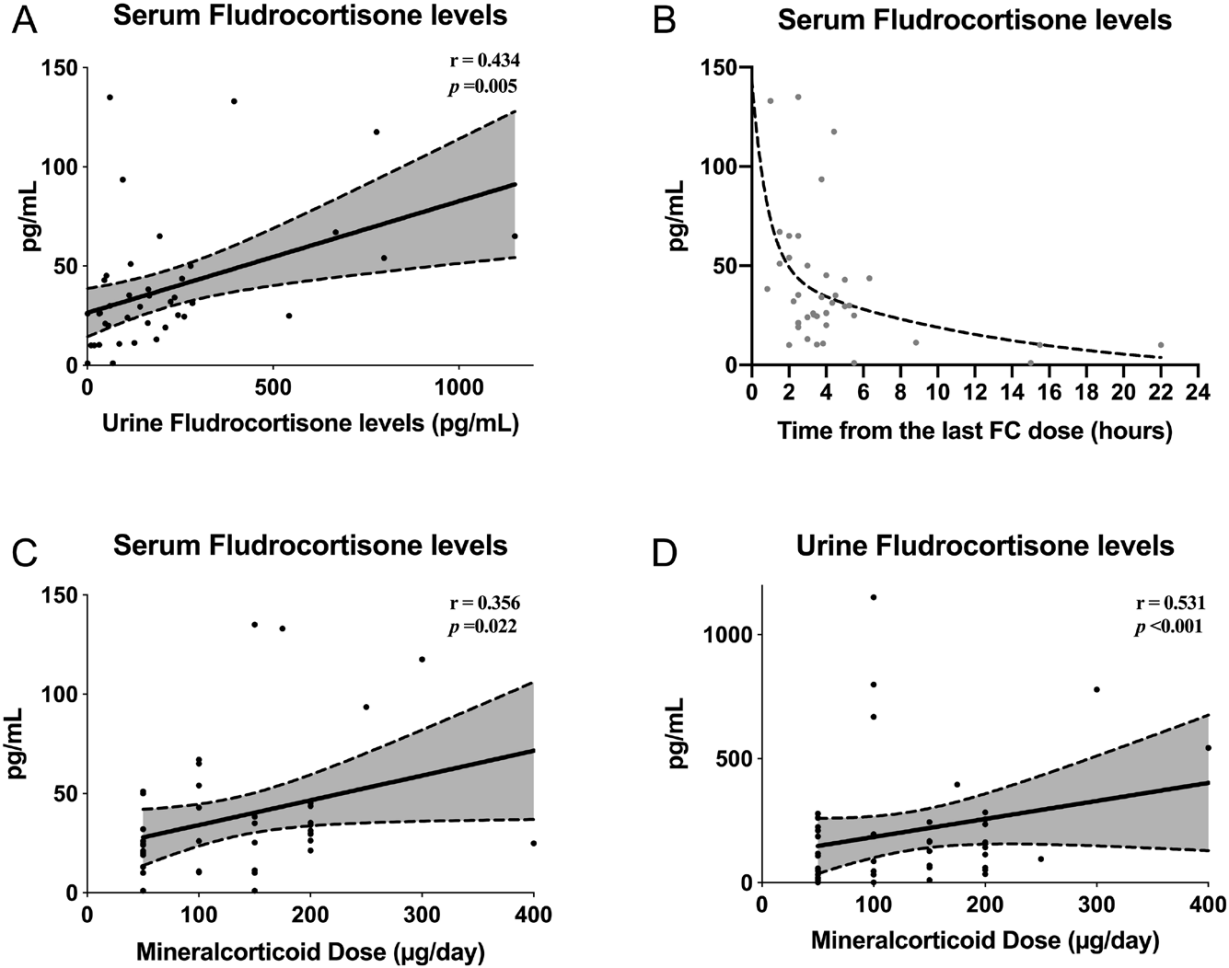
Univariate analysis for serum and urine FC levels in a cohort of 41 patients with primary adrenal insufficiency. The dashed line in (B) refers to a two-phase exponential decay equation derived from serum FC levels at different timepoints. Grey areas between the dashed lines in (A), (C) and (D) refer to the 95% confidence intervals of the regression lines.

Univariate analysis across the whole cohort demonstrated a tight positive relationship between sFC and uFC levels (*r* = 0.434, *P* = 0.005) (Fig. 1A). Endorsing the accuracy of the adherence data, sFC showed an inverse correlation with the time from the last FC dose (*r* = −0.355, *P = *0.023) (Fig. 1B), with a peak in serum concentration 1-h after administration and an estimated half-life of 1 to 3 h which is consistent with published data (32, 33, 34).

**Figure 2:**
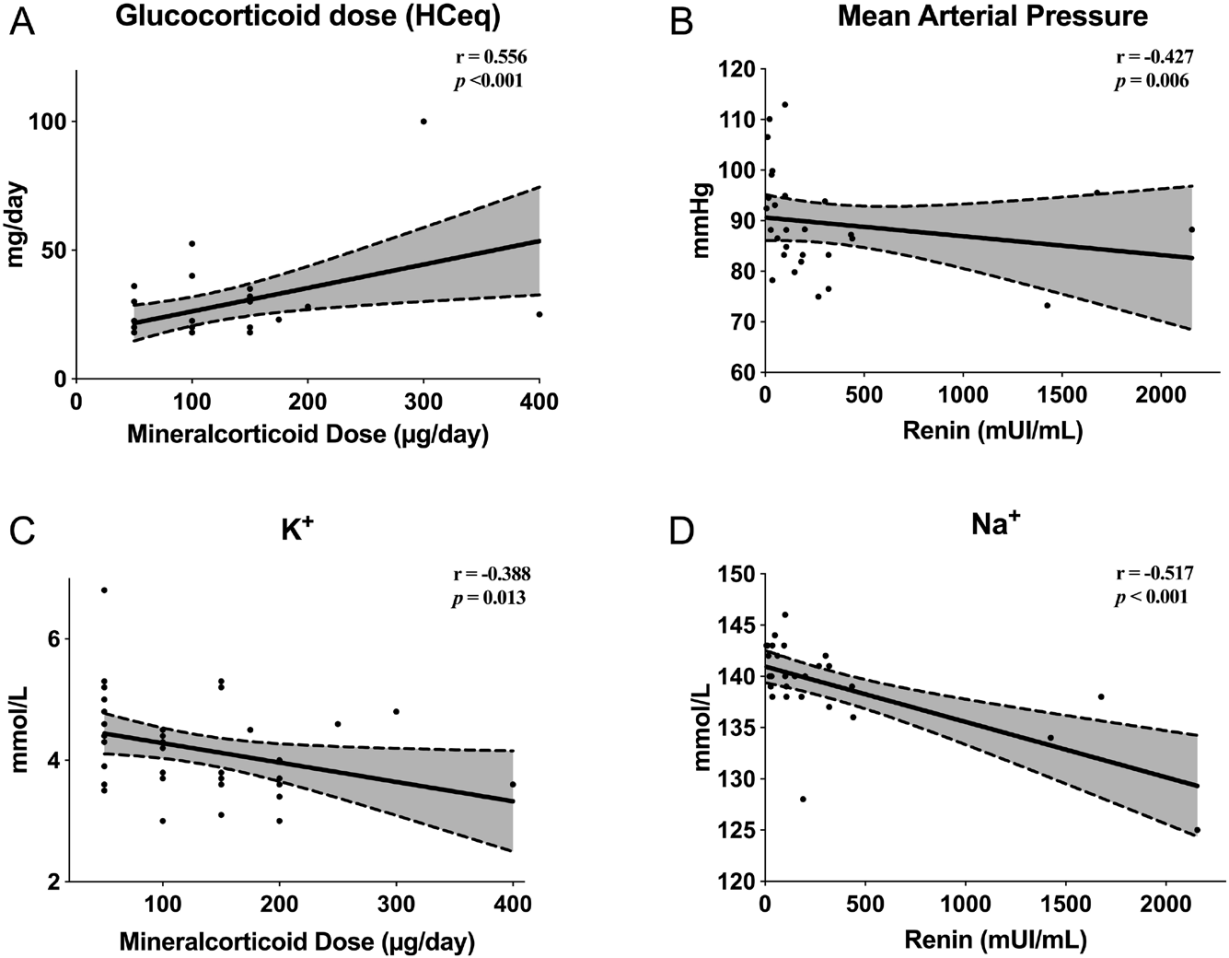
Univariate analysis for clinical and biochemical variables in a cohort of 41 patients with primary adrenal insufficiency. Grey areas between the dashed lines refer to the 95% confidence intervals of the regression lines.

In a univariate model, both sFC and uFC were positively related to total daily MC dose (sFC: *r* = 0.356, *P* = 0.022; uFC: *r* = 0.531, *P* < 0.001) (Fig. 1C and D). There were no univariate relationships between sFC or uFC and PRC. However, there was a strong positive correlation between total daily GC and MC doses (*r* = 0.556, *P* < 0.001) and an inverse relationship between MAP and PRC (*r* = −0.427, *P* = 0.006) (Fig. 2A andB). In agreement with our previous observations (24), there was a negative association between total MC dose and potassium levels (*r* = −0.388, *P* = 0.013) and between sodium and PRC levels on univariate analysis (*r* = −0.517, *P* < 0.001) (Fig. 2C and D). A multiple regression model assessing the ability of biochemical variables in predicting total daily MC dose is reported in the supplementary data. After excluding one patient taking diuretics, all the analyses were repeated and the results were unaffected (data not shown).

### Relationships between therapy adherence and clinical parameters

Eight patients (20%) missed at least one dose of their GC replacement therapy in the preceding 7 days; Four patients (10%) declared missing at least one dose of both GC and FC whilst one patient missed a single FC dose in isolation. In total, 13 (32%) patients were classified as ‘non-adherent’. MC dose frequency did not affect patients’ adherence (*P* = 0.744). Among the 26 patients treated with more than one dose of GC per day, 11 showed non-adherence (*P* = 0.084). There were no differences in adherence between patients taking HC or MRHC (*P* = 0.545).

Non-adherent patients were younger (median age 29 years, IQR 25–33 vs49 years, IQR 32–66, *P* = 0.009), were mainly affected by SW-CAH (ten SW-CAH vs three AD, *P <* 0.001), were prescribed higher FC (200 μg/day, IQR 150–200 vs 100 μg/day, IQR 50–150, *P* < 0.001) and higher GC replacement doses (25 mg/day HCeq, IQR 23–36 vs 22.5 mg/day, IQR 18–30, *P* = 0.031) and had lower potassium levels (3.6mmol/L, IQR 3.4–4.0 vs 4.3mmol/L, IQR 3.7–4.8, *P* = 0.033). There were no differences in sodium levels, PRC or MAP (Table 2). Specifically with regard to FC replacement, there were no differences in sFC (*P* = 0.622) and uFC (*P* = 0.127) when comparing FC adherent (*n* = 36) and non-adherent (*n* = 5) patients. In patients with CAH (*n* = 17), biochemical makers of disease control were lower in the GC adherent (*n* = 7) compared to non-adherent (*n* = 10) patients (Androstenedione (median, IQR): 2.4 ( 1.0–14.9) nmol/L vs13.3 (9.8–34.43), *P* = 0.04; 17OH-progesterone: 2 (1–90) nmol/L vs 55 (36–119), *P* = 0.07) (Table 3).

**Table 2:** Characteristics of 41 patients with PAI enrolled into the study, stratified for patients’ adherence.

	*Adherent* (*n* = 28)	*Non-adherent* (*n* = 13)
Age (years)	49 (32–66)	29 (25–33)^a^
Male % (*n*)	57 (16)	69 (9)
Female % (*n*)	43 (12)	31 (4)
Total daily MC dose (μg/day)	100 (50–150)	200 (150–200)^a^
Patients with SW-CAH, % (*n*)	25 (7)	77 (10)
Patients with AD, % (*n*)	75 (21)	23 (3)
Total daily GC dose (mg/day, HCeq)	22.5 (18–30)	25 (23–36)^a^
Hydrocortisone % (*n*)	82 (23)	31 (4)
Prednisolone % (*n*)	18 (5)	69 (9)
BMI (kg/m²)	25.7 (21.8–30.3)	27.0 (20.8–31.9)
SBP (mmHg)	120 ± 17	119 ± 12
DBP (mmHg)	74 ± 9	67 ± 8
MAP (mmHg)	89 ± 10	84 ± 9
Na⁺ (mmol/L)	140 (138–142)	140 (139–142)
K⁺ (mmol/L)	4.3 (3.7–4.8)	3.6 (3.4–4.0)^a^
PRC (μIU/mL)	107.3 (36.4–315.3)	164.6 (95.0–313.7)
17-OH-progesterone (nmol/L)^b^	2.0 (1.0–90.0)	55.0 (35.6–119.0)
Androstenedione (nmol/L)^b^	2.4 (1.0–14.9)	13.3 (9.8–34.43)
sFC (pg/mL)	25.1 (10.8–50.7)	34.2 (27.9–44.4)
uFC (pg/mL)	115.0 (35.9–256.7)	163.0 (56.3–245)

Data are shown as mean ± s.d. or median (IQR) as appropriate for distribution of data.

^a^ *P* < 0.05 vs adherent; ^b^Only patients with CAH (*n* = 17).

AD, Addison’s disease; BMI, body mass index; DBP, diastolic blood pressure; GC, glucocorticoid; HCeq, equivalent dose of hydrocortisone; K+, potassium levels; MAP, mean arterial pressure; MC, mineralocorticoid; Na+, sodium levels; PRC, plasma renin concentration; SBP, systolic blood pressure; sFC, serum fludrocortisone levels; SW-CAH, salt-wasting congenital adrenal hyperplasia; uFC, urinary fludrocortisone levels.

**Table 3:** Multiple regression model in 41 adults with primary adrenal insufficiency. The dependent variable was total daily mineralocorticoid dose (MC dose). The independent variables were serum sodium (Na^+^), serum potassium (K^+^), mean arterial pressure (MAP), plasma renin concentration (PRC), body mass index (BMI), total daily hydrocortisone-equivalent glucocorticoid dose (dGC), serum fludrocortisone levels (sFC), urine fludrocortisone levels (uFC), GC formulations and patients’ adherence. Significant *P* values are highlighted in bold and with superscript lowercase letters (*r* ^2^ = 0.58,*P* = 0.009^a^).

Independent	Dependent: total daily MC dose			
	*B*	95% CI lower bound	95% CI upper bound	*P*
Na^+^ (mmol/L)	−6.350	−13.536	0.835	0.081
K^+^ (mmol/L)	−32.086	−66.979	2.807	0.070
MAP (mmHg)	−0.258	−2.687	2.172	0.829
PRC (mIU/L)	0.012	−0.055	0.078	0.722
BMI (kg/m^2^)	1.029	−2.719	4.777	0.576
dGC (mg of HCeq)	1.022	−1.462	3.507	0.404
sFC (pg/mL)	15.615	−43.004	74.234	0.588
uFC (pg/mL)	9.593	−34.111	53.298	0.655
GC formulation	6.464	−57.608	70.537	0.837
Patient adherence	−50.676	−97.630	−3.722	**0.036^a^**

Baseline correlation analyses were repeated excluding all ‘non-adherent’ patients and confirmed the strong relationship between sFC and uFC levels (*r* = 0.578, *P* < 0.001). In addition, the relationship between total daily GC and MC doses persisted (*r* = 0.435, *P* = 0.021), as did the inverse relationship of PRC to sodium levels (*r* = −0.585, *P* = 0.001) and the positive association of PRC with potassium levels (*r* = 0.553, *P* = 0.002) (Supplementary Fig. 1, see section on supplementary materials given at the end of this article).

A multiple regression model was constructed to identify variables that could predict total daily MC dose and included all measured clinical and biochemical variables (sFC, uFC, Na^+^, K^+^, MAP, PRC, BMI, total daily GC dose and GC formulation) as well as data on patient’s treatment adherence. The model was significant (*r* ^2^ = 0.58, *P* = 0.009), there was no relationship between sFC or uFC with total daily MC dose, suggesting that they are unhelpful in guiding total daily MC dose. Treatment adherence was the only variable able to predict total daily MC dose (*B* = −50.676, *P* = 0.036). All the computed and relative coefficients generated by the models are summarized in Table 3. Confirming the regression analysis, ‘non-adherent’ patients were found to have higher chance to be prescribed with FC dose higher than or equal to 200 μg/day (OR 18.87, *P* < 0.001).

## Discussion

In this study, we have assessed the utility of measuring sFC and uFC levels to help guide MC replacement. Whilst there were robust relationships between sFC, uFC and total MC replacement dose, there were no relationships with clinically important variables including electrolytes and blood pressure. As a result, there appears to be little additional value in the routine measurement of sFC or uFC in clinical practice.

Current guidance suggests that MC dose titration should be based on clinical assessments (blood pressures in supine and standing positions to rule out orthostatic dysregulation, salt craving and peripheral oedema) and biochemical variables (electrolytes, PRC) (11, 35). However, a significant proportion of patients replaced with FC still complain of MC deficiency-related symptoms (36, 37) suggesting that current methods of assessment of MC replacement therapy maybe sub-optimal.

Achieving electrolyte levels in the normal range, a normal blood pressure without postural hypotension (12, 37) and PRA in the upper limit of normal has been postulated to represent the targets to demonstrate adequacy of MC replacement (38), but this has not been uniformly accepted (22, 23, 39, 40). The recommendations concerning the role of renin in FC titration derive from historical studies, with a low number of patients and, importantly have used PRA as opposed to PRC (25, 26, 38). More recently, the measurement of PRA has been replaced with PRC; the PRA assay is time-consuming and affected by substrate (angiotensin) availability (12, 41) which might be altered during oestrogen and GC treatment. A comparison of the clinical utility of PRA vs PRC in PAI has never been performed and assessment of PRC seems to be more reliable than PRA in the management of PAI (26). A recent single-centre study on 193 patients with PAI showed that renin together with serum electrolytes relates to MC dose, but the study included both PRC and PRA evaluations (27). In contrast, we reported the results of a retrospective ‘real-life’ observational study conducted on 280 patients with PAI in which routine monitoring of BP and serum electrolytes were more informative than PRC to titrate MC dose (24). It is important to note that studies such as this maintain the integrity of the context in which medical care is provided (42, 43), as distinct from that in tightly controlled randomized studies and perhaps make them more relevant to everyday clinical practice.

In the current study, we have also demonstrated a dissociation between PRC and MC daily dose. Indeed, the total daily MC dose was only inversely related to serum potassium levels. Higher PRC was associated with lower sodium levels and MAP, confirming PRC as a marker of plasma volume and sodium state (44). These data, taken together with those in the published literature, highlight the complex interactions between renin, blood pressure and circulating electrolytes in MC-deficient patients and this, combined with the lack of an internationally accepted standard reference range, makes their interpretation challenging (11, 24).

Adherence to medication is commonly overlooked in ‘real-life’ clinical research, retrospective studies as well as in clinical trials (28, 29). The data from this study indicate that it is a major factor in determining the total daily MC dose.

In patients with chronic diseases requiring long-term treatments, poor medication adherence is particularly prevalent (45). Medication adherence can be measured indirectly, through self-reports, pill counts, electronic monitoring or pharmacy prescription data, or directly by measuring the concentration of medications in blood or urine (46). Difficulties in the accurate assessment of adherence in clinical settings are well-described (47), and self-reports often overestimate adherence (46, 48). However, this remains the most useful and reliable method in the adult clinical setting (46, 49), since physician estimates of patients’ adherence are less informative (50); discordance rates of approximately 30% in patients with CAH have been reported (48). In the current study, we questioned whether sFC and uFC might reflect adherence with MC treatment, although we did not find any differences between ‘adherent’ and ‘non-adherent’ patients. This might be related to the small number of patients within the study but also to the pharmacokinetics of FC, where low levels might only be expected if a patient missed an MC dose less than 18 h before blood or urine sampling.

In total, we found 32% of our patients to be ‘non-adherent’ with FC and/or GC treatment. This observation does raise concerns, especially in this patient group who are at significant serious risk of adrenal crisis and the associated morbidity and mortality. We assessed treatment adherence in two different conditions (AD and SW-CAH). Little is known about medication adherence in adults with PAI; however, endorsing our findings, two published studies have shown non-adherence rates in adults with CAH ranging from 26 to 36% (48, 51). In our cohort, compared to those with AD, patients with SW-CAH were younger and many were in a transition age range between adolescence and adulthood. Deficiencies in treatment adherence during this transition period are well recognised in patients living with CAH (51, 52) and this may offer an explanation as to why in our cohort patients with CAH were prescribed higher FC dose compared to patients with AD. Similarly, as expected, we found that ‘non-adherent patients’ were prescribed higher FC and GC doses, despite showing lower potassium levels. This has important clinical consequences as ‘non-adherent’ patients had a 20 times higher risk of receiving FC doses in excess of those recommended in current guidelines (200 μg/day). Although our cohort included both GC and MC ‘non-adherent’, it is unlikely that non-adherence with FC treatment will have affected these results. This is supported by previous data confirming that, due to the escape phenomenon from the salt-retaining effect which takes place during prolonged administration of MCs, a new steady state is often obtained at least after 1 week of FC dose change (53), and therefore, changes in electrolyte levels and PRA/PRC cannot be seen earlier than 1 or 2 weeks (23) although our adherence assessment over the preceding 7 days is likely to reflect their broader, longer-term adherence. Importantly, unlike GC replacement, patients can omit their FC and feel well for several days before the body's sodium reserve is depleted (44).

Through the incorporation of adherence assessment into our models to predict MC dosing, we have demonstrated that its impact is strong affecting the reliability of clinical and biochemical variables. In addition, by refining our analysis and excluding ‘non-adherent’ patients, we found significant associations reflective of underlying physiology including the relationships between PRC and electrolytes levels, and GC with MAP. Current guidance for the treatment of PAI state that ‘…assessment of renin should be done in response to changes in clinical status or if compliance is in question…’(11). Whilst we would agree that renin can certainly be useful in cases of very poor adherence (as a diagnostic marker of MC insufficiency), here we show that adherence appears to affect most (if not all) the variables commonly used in clinical practice. We therefore suggest that treatment adherence in PAI should be evaluated first before all the other tools for titration of GC and FC therapy are taken into consideration in the process of tailoring replacement doses.

There are several limitations to the current study and analysis. The sample size was relatively small and as a result, our findings may not be representative. PRC was measured, without a standardised posture protocol, in two different centres and a direct comparison between the two assays was not performed. No data about FC treatment duration and administration in relation to food, nor about salt craving were collected, although such data are difficult to quantify and the assessment of adherence was limited to a small number of specific questions. uFC levels were measured in spot urine samples which, albeit less onerous for participants, might be dependent on hydration status, duration and volume of the collection as well as residual bladder volume. Moreover, no data about urine electrolytes (notably sodium and potassium) were collected. Whilst there are studies demonstrating the reliability of urine spot samples, our results should be confirmed using 24-h urine measurement of FC and electrolytes. The observational nature of our study design is clearly an additional limitation; however, it does reflect real-life clinical practice and is representative of the challenges faced by clinicians managing patients with PAI.

In conclusion, our study provides real-life insights into addressing the clinically important challenge of optimizing MC replacement in patients with PAI. Whilst we demonstrate a lack of utility of the measurement of sFC and uFC, this study highlights the crucial role of adherence with medication and the need for this to incorporate as part of the routine clinical care assessment in patients with PAI. Only once the degree of adherence is known, can a meaningful interpretation of other relevant clinical variables including blood pressure and electrolytes and perhaps PRC be made.

## Supplementary Materials

Supplementary Material

## Declaration of interest

The authors declare that there is no conflict of interest that could be perceived as prejudicing the impartiality of the research reported.

## Funding

The study was supported by an MRC programme grant awarded to JWT ref. MR/P011462/1.
