# Supplementary material for: Assessing treatment adherence is crucial to determine adequacy of mineralocorticoid therapy
Source: Endocr Connect. 2023 Aug 2;12(9):e230059. doi: 10.1530/EC-23-0059 (PMC10448575; doi:10.1530/EC-23-0059)
Supplement: Supplementary Material [file supplementary_material.pdf]

Supplementary file for EC-23-0059.

Title:

Assessing treatment adherence is crucial to determine adequacy of mineralocorticoid therapy.

Authors

Riccardo Pofi[1], *Ilaria Bonaventura*[2], *Joanne Duffy*[3], *Zoe Maunsell*[4], *Brian Shine*[4], *Andrea M. Isidori*[2], *Jeremy W Tomlinson*[1].

Affiliations

[1]*Department of Endocrinology, Oxford Centre for Diabetes, Endocrinology and Metabolism and NIHR Oxford Biomedical Research Centre, Churchill Hospital, University of Oxford, Oxford, OX37LE, UK;*

[2]*Department of Experimental Medicine, Sapienza University of Rome, Viale Regina Elena 324, 00161, Rome, Italy;*

[3]*Department of Clinical Chemistry and Immunology, Heartlands Hospital, Birmingham, B9 5SS, UK*

[4]*Department of Clinical Biochemistry, Oxford University Hospitals NHS Foundation Trust, Oxford, UK*

Multiple regression model with only biochemical variables

A multiple regression model was constructed to identify variables that could predict total daily MC dose and included all measured clinical and biochemical variables (sFC, uFC, Na^+, K^+, MAP, PRC, BMI, total daily GC dose and GC formulation). Whilst the analysis was significant (r^2=0.58, *p*=0.004), there was no relationship between sFC or uFC with total daily MC, suggesting that they are unhelpful in guiding total MC daily dose. The only clinical variable to predict total daily MC dose was the potassium (*B*= 31.457, *p*=0.051). All the computed and relative coefficients generated by the models are summarized in *supplementary table 1*.

Supplementary table 1

Multiple regression model in 41 adults with primary adrenal insufficiency. The dependent variable was total daily mineralocorticoid dose (MC dose). The independent variables were serum sodium (Na^+), serum potassium (K^+), Mean Arterial Pressure (MAP), plasma renin concentration (PRC), Body Mass Index (BMI), total daily hydrocortisone-equivalent glucocorticoid dose (dGC), serum fludrocortisone levels (sFC), urine fludrocortisone levels (uFC) and GC formulation. Significant *p* value are highlighted in bold and with asterisk.

r^2 = 0.58, p*=0.004	**Dependent: Total daily MC Dose**			
Independent	***B***	***95% CI lower bound***	***95% CI upper bound***	***p***
Na^+ (mmol/L)	-6.020	-12.584	0.544	0.071
**K^+ (mmol/L)**	**-31.457**	**-63.461**	**-0.546**	**0.051***
MAP (mmHg)	-0.211	-2.393	1.970	0.843
PRC (mIU/L)	0.018	-0.043	0.080	0.538
BMI (kg/m^2)	0.391	-3.064	3.846	0.817
dGC (mg of HCeq)	1.174	-1.109	3.458	0.299
sFC (pg/mL)	10.994	-42.955	64.942	0.678
uFC (pg/mL)	27.397	-12.930	67.723	0.174
GC formulation	34.655	-20.329	89.639	0.206

Supplementary figure 1. Baseline correlations in 28 patients with PAI who were adherent with replacement therapy. Grey areas between the dashed lines in figures a), c) and d) refer to the 95% confidence intervals of the regression lines.

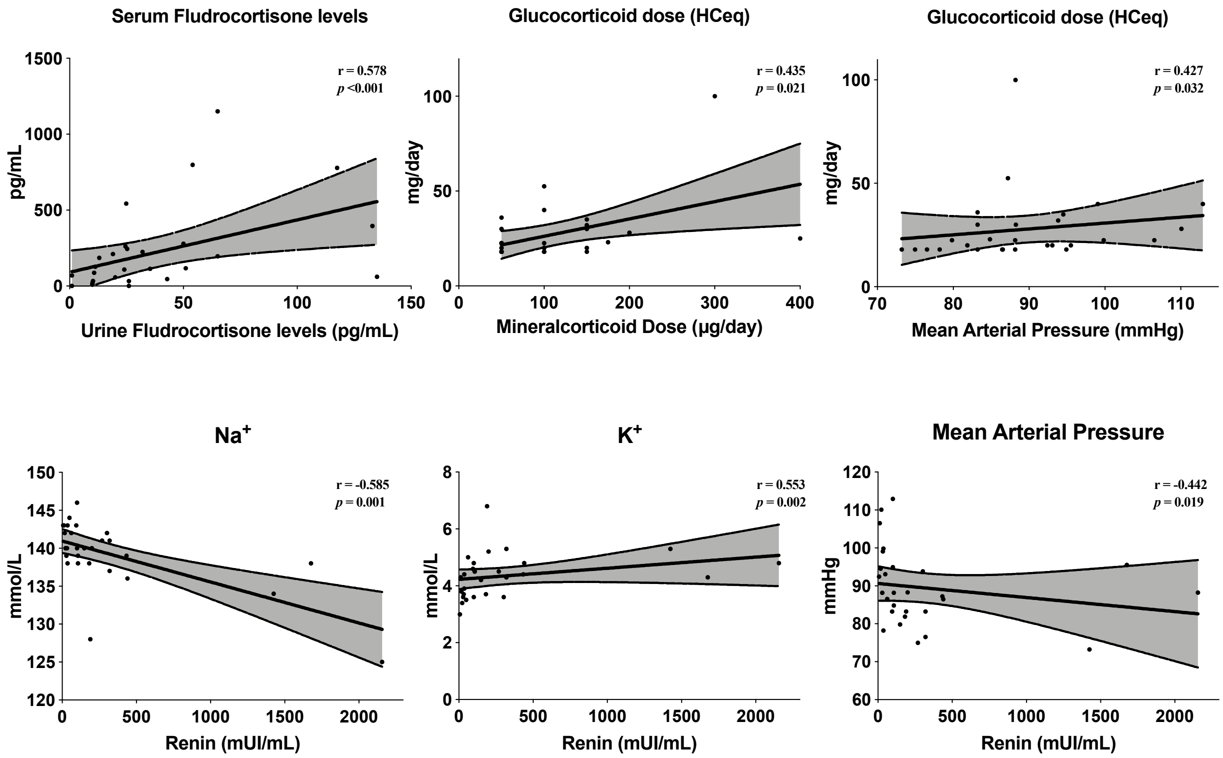

Supplementary figure 2. Copy of the questionnaire for the assessment of treatment adherence in patients with primary adrenal insufficiency.

Patient details:

Date seen in clinic: ………………………………………………………

Clinic blood pressure: *Lie:* *Stand:*

Postural blood pressure (if recorded)

Height (m): ……………………………………………………………

Weight (kg): ……………………………………………………………

BMI (kg/m^2): ……………………………………………………………

Regular fludrocortisone regimen:

Dose: ……………………………………………………………………..

Frequency: ……………………………………………………………

Date of last fludrocortisone administration: ……………………

Time of last fludrocortisone administration: …………………...

Dose of last fludrocortisone administration: …………………..

Number of missed doses in the last week: ………………………

Is you patient on glucocorticoid replacement therapy? Yes No

Which glucocorticoid? ………………………………………………

Dose: ……………………………………………………………………

Frequency: …………………………………………………………..

Date of last glucocorticoid administration: …………………….

Time of last glucocorticoid administration: …………………....

Dose of last glucocorticoid administration: ……………………

Number of missed doses in the last week: …………………….
